# Crystal structures of tolfenamic acid polymorphic forms I and II with precise hydrogen-atom positions for nuclear magnetic resonance studies

**DOI:** 10.1107/S2056989020010841

**Published:** 2020-08-11

**Authors:** Helen Blade, Charles D. Blundell, Iñigo J. Vitorica-Yrezabal

**Affiliations:** a Oral Product Development, Pharmaceutical Technology & Development, Operations, AstraZeneca, Macclesfield, SK10 2NA, United Kingdom; b C4X Discovery, 53 Portland Street, Manchester, M1 3LD, United Kingdom; c School of Chemistry, The University of Manchester, Oxford Road, Manchester, United Kingdom

**Keywords:** polymorph, redetermination, Hirshfeld atom refinement, nitro­gen–hydrogen bond length, crystal structure

## Abstract

The structures of tolfenamic acid [TFA; 2-(3-chloro-2-methyl­anilino)benzoic acid, C_14_H_12_ClNO_2_] polymorph forms I and II have been redetermined [compare Andersen *et al.* (1989 ▸). *J. Chem. Soc., Perkin Trans. 2*, pp. 1443–1447] with improved precision using high-resolution X-ray diffraction data and Hirshfield atom refinement in order to better define both hydrogen-atom locations and their associated bond lengths. Covalent bond lengths to hydrogen were found to be significantly longer throughout both structures, especially for the anilino H atom, which is involved in an important intra­molecular N—H⋯O hydrogen bond to the carb­oxy­lic acid group. This hydrogen bond is shown to clearly perturb the electron density around both oxygen atoms in the latter group. The extended structures of both polymorphs feature carb­oxy­lic acid inversion dimers. These structures provide an improved foundation for nuclear magnetic resonance studies in both solution and the solid state.

## Abstract 2

The structures of tolfenamic acid polymorph forms I and II have been redetermined with improved precision of the hydrogen-atom positions by Hirshfeld atom refinement to provide improved data for solid- and solution-state nuclear magnetic resonance studies.

## Chemical context   

Tolfenamic acid (TFA; 2-[(3-chloro-2-methyl­phen­yl)amino]­benzoic acid; C_14_H_12_ClNO_2_) is a non-steroidal anti-inflammatory drug (NSAID). It is frequently used as a model for crystallography studies because it displays inter­esting polymorphism, with eight forms identified to date (Andersen *et al.*, 1989 ▸; López-Mejías *et al.*, 2009 ▸; Case *et al.*, 2018 ▸). Moreover, its small size and simple crystal structures permit timely computational calculations, which is advantageous for studies investigating its behaviour by nuclear magnetic resonance (NMR).
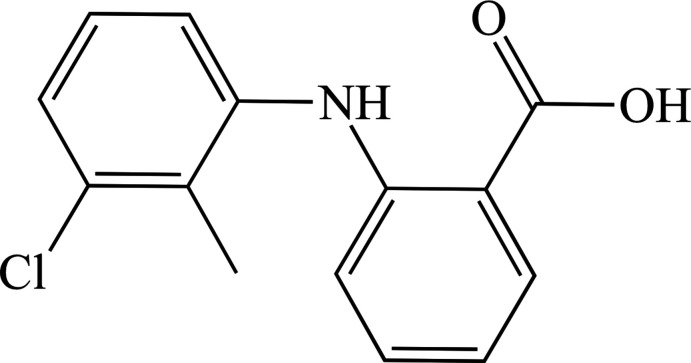


The two most common polymorphs of TFA (forms I and II) differ principally in the dihedral angles between the phenyl rings, giving different overall conformations and attendant packing arrangements (Andersen *et al.*, 1989 ▸). A number of experimental and theoretical studies have been performed on TFA to explore the origin of this (see *e.g.* Ang *et al.*, 2016 ▸; Du *et al.*, 2015 ▸; Mattei & Li, 2012 ▸, 2014 ▸; Mattei *et al.*, 2013 ▸). Both form I and form II are easily prepared in sufficient amounts and purity under standard laboratory conditions to permit solid-state NMR studies and they are readily distinguishable by their colour (form I, white; form II, yellow) (Andersen *et al.*, 1989 ▸). TFA is soluble in a variety of crystallization solvents and is suitable for having its conformational behaviour precisely characterized by solution-state NMR methods (Blundell *et al.*, 2013 ▸).

Our motivation for the present study was the desire to generate high resolution structures of both forms I and II in which the locations of the hydrogen atoms and their attendant bond lengths were precisely resolved. Accurate hydrogen positions are important for both solid- and solution-state NMR studies because ^1^H is a chief nucleus for acquiring experimental data through and a variety of experimental observables depend sensitively on bond lengths to hydrogen (*e.g.* chemical shifts of hydrogen atoms involved in hydrogen bonding, see Siskos *et al.*, 2017 ▸; residual dipolar couplings, see Lipsitz & Tjandra, 2004 ▸). Structures with precise hydrogen positions therefore provide more robust starting points for density functional theory (DFT) calculations of NMR observables in the solid-state and, in the solution state, a better mean-average geometry to found conformational analysis upon.

Accordingly, large single crystals (needles 0.5–1 mm in length) of forms I and II were grown that diffracted to *d* ≃ 0.48 Å (2θ_max_ = 95.5°, *T* = 100 K) and 0.53 Å (83.6°, 150 K), respectively with Mo *K*α radiation. The structures of each form were solved and refined using Hirshfeld atom refinement, which determines the structural parameters from single crystal X-ray diffraction data by using an aspherical atom partitioning of *ab initio* quantum mechanical mol­ecular electron densities (Capelli *et al.*, 2014 ▸). Significantly for our purpose, the precision of the determined bond lengths and anisotropic displacement parameters for the hydrogen atoms calculated with Hirshfeld atom refinement with data of this resolution is comparable to that from neutron diffraction measurements (Fugel *et al.*, 2018 ▸).

## Structural commentary   

**Figure 1:**
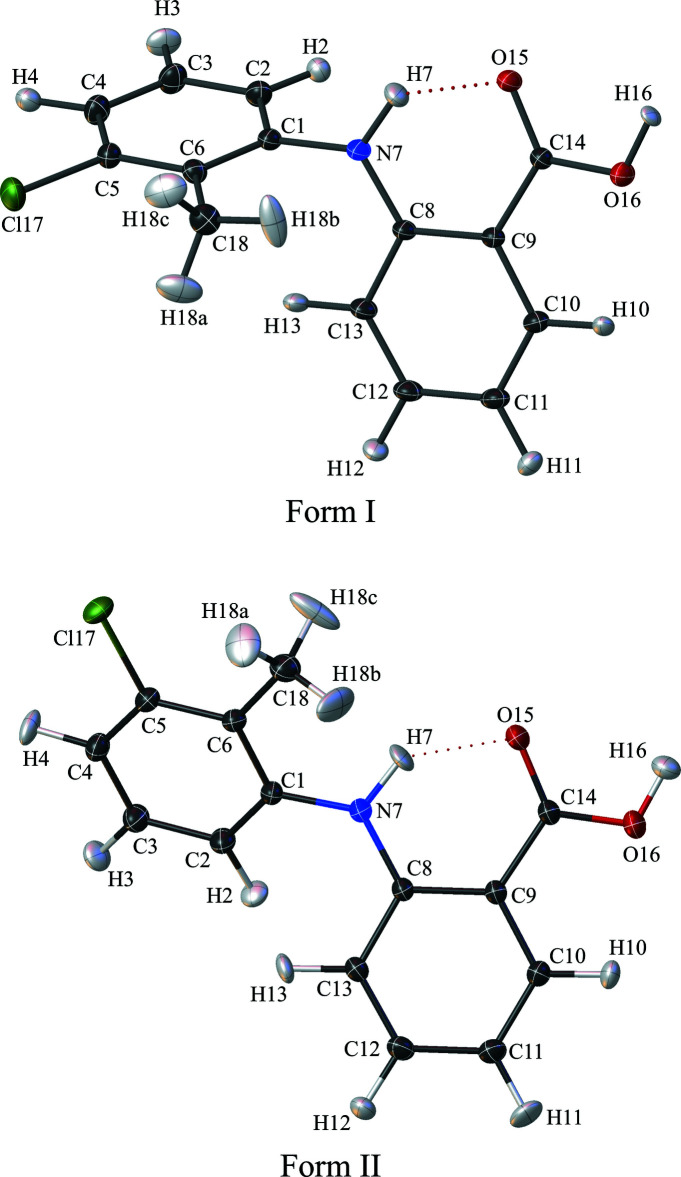
The mol­ecular structures of TFA in form I and form II, with atom labelling. The inter­nal N7—H7⋯O15 hydrogen bond is indicated with a dotted line. Displacement ellipsoids are drawn at the 50% probability level.

The crystal structure determination of forms I and II was achieved (Fig. 1 ▸). Both forms are monoclinic, with form I in space group *P*2_1_ */c* and form II in *P*2_1_ */n*; both structures comprise *Z′* = 1 and *Z* = 4 and form an inter­nal hydrogen bond between N7—H7 and O15 with very similar geometry (Tables 1 ▸ and 2 ▸); this inter­nal hydrogen bond makes the amino­benzoic acid group adopt an essentially planar conformation. The chief difference between the two forms is the dihedral angle between the C1–C6 and C8–C13 phenyl rings, being 72.82 (4)° for form I and 44.34 (3)° for form II. This is also seen in the torsion angle C8—N7—C1—C6, with values of 74.34 (12) and −143.00 (6)° for forms I and II, respectively (in the crystal an equal number of mol­ecules have the opposite sign for these torsion angles). Additionally, the C8—N7—C1 angle also differs somewhat [form I 123.97 (7); form II 129.34 (5)°]. The methyl group torsion angle differs between the two structures, with form I having one hydrogen atom almost coplanar with the 3-chloro-2-methyl­phenyl ring and form II having one hydrogen atom orthogonal to it. The displacement parameters of the ellipsoids show that the methyl groups in both structures display greater motion relative to the rest of their structures.

## Supra­molecular features   

**Figure 2:**
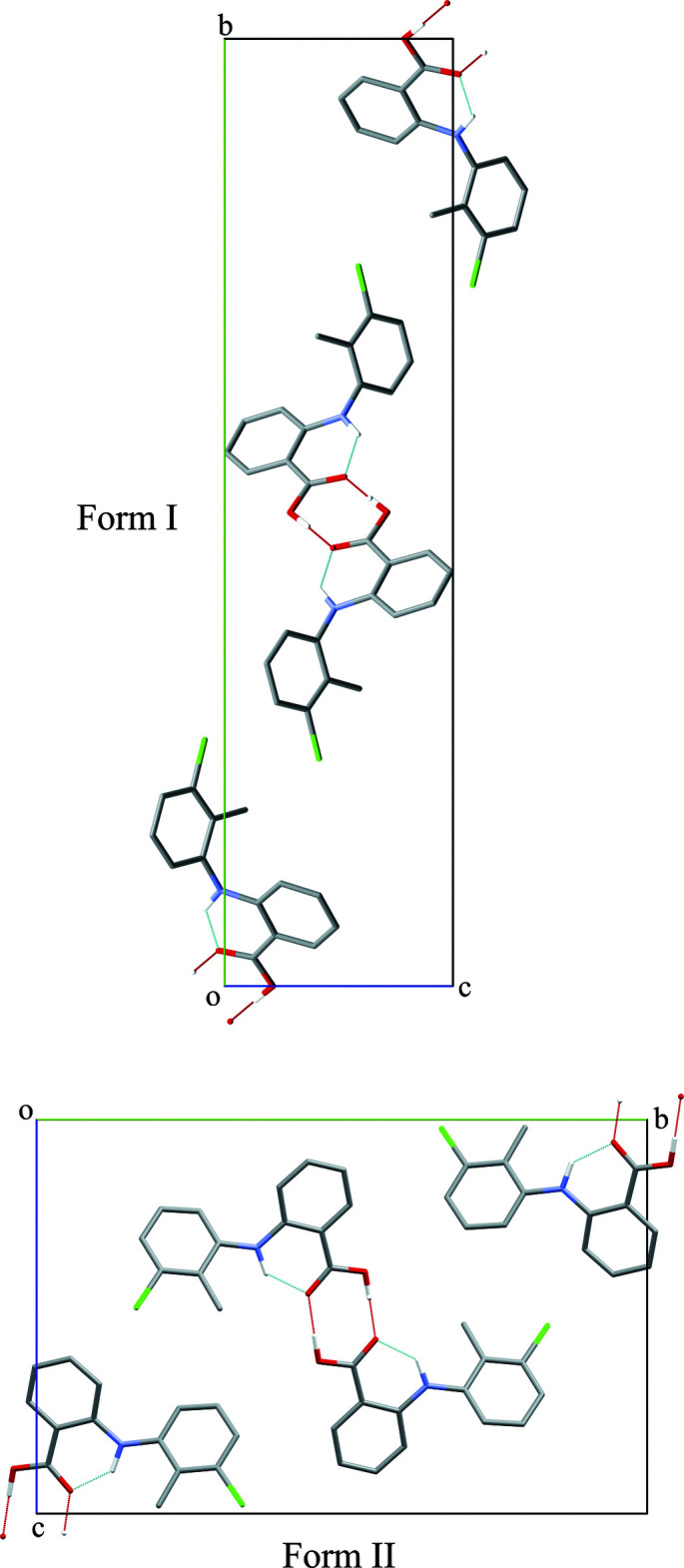
Crystal structures of TFA form I and form II showing their inversion-dimer pairs and differing packing arrangements (both viewed along the *a* axis). Hydrogen bonds are indicated with dotted lines. For clarity, only polar hydrogen atoms have been included.

**Figure 3:**
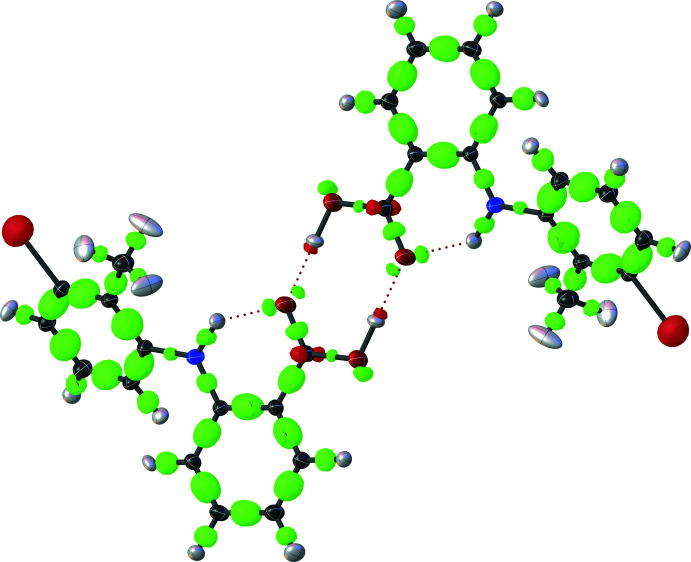
Crystal structure of TFA form II, showing the hydrogen-bond network (red dotted lines) and the deformation of the electron density calculated by *TONTO* (Capelli *et al.*, 2014 ▸). Negative electron density is shown with green surfaces and positive electron density is shown with red (threshold level −0.25, Res/Å). Ellipsoids of carbon atoms are shown in grey, nitro­gen blue, oxygen red, hydrogen white and chlorine green.

The packing arrangement for both forms are shown in Fig. 2 ▸. In both structures, O—H⋯O hydrogen-bonding inter­actions between the carb­oxy­lic acid groups on pairs of TFA mol­ecules result in the formation of symmetrical hydrogen-bonded dimers that are related by an inversion centre (Tables 1 ▸ and 2 ▸; Figs. 3 ▸ and 4 ▸).

The structure determination has precisely resolved not only hydrogen-atom positions but also the shape and positions of the electron density corresponding to the mol­ecular orbitals throughout the structure (Figs. 3 ▸ and 4 ▸). Very clear differences between the carboxyl­ate oxygen atoms in the dimer hydrogen-bonding motif are now apparent. Bond C14—O15 has more electron density than C14—O16, revealing its greater double-bond character. Oxygen atom O15 accordingly adopts an *sp^2^* geometry, with its two lone-pairs clearly located in the expected co-planar positions (*i.e*., at ±120° from the C15—O16 bond); one lone pair accepts the intra­molecular hydrogen bond from H7 while the other receives an inter­molecular hydrogen bond from H16. Atom O16 in contrast has a covalent bond to H16, for which electron density is clearly visible. Despite the apparently dominant double-bond character of C14—O15, O16 is inter­estingly not simply forming a purely single bond with C14 and adopting an *sp^3^* hydridization state: the typical positions of the two *sp^3^* mol­ecular orbitals projecting away from and above and below the carboxyl­ate plane have smeared into a single lobe of density with a significant amount of coplanar (*i.e.*, *sp^2^*-like) electron density.

In short, the typical equivalence of the O atoms in the carb­oxy­lic acid has been significantly perturbed by the N7—H7⋯O15 intra­molecular hydrogen bond. These electronic perturbations should be remembered when mol­ecules containing carb­oxy­lic acid groups are being designed to inter­act with protein targets via hydrogen bonding.

## Database survey   

A search for crystal structures containing TFA explicitly in its protonated state was performed within the Cambridge Structural Database (CSD version 5.41, update November 2019; Groom *et al.*, 2016 ▸). There are eight polymorphs of pure TFA (CSD reference codes KAXXAI, KAXXAI01–07, Andersen *et al.*, 1989 ▸; López-Mejías *et al.*, 2009 ▸; Case *et al.*, 2018 ▸) and six co-crystal forms (EXAQIE, Fábián *et al.*, 2011 ▸; SIMDOK/01 & SIMFUS/SIMGAZ/SIMGED, Case *et al.*, 2018 ▸; UZUZIA & UZUZOG, Bouanga Boudiombo & Jacobs, 2016 ▸; XOWKAX/01, Surov *et al.*, 2015 ▸, Wittering *et al.*, 2015 ▸). In all cases, the inter­nal hydrogen-bond equvialent to N7—H7⋯O15 in the present structures is present and the hydroxyl hydrogen atom is attached to the corresponding O16 equivalent. The C8—N7—C1—C6 torsion angle ranges from 75.0 to 138.4° in the pure forms and from 76.1 to 156.9° in the co-crystals.

**Figure 5:**
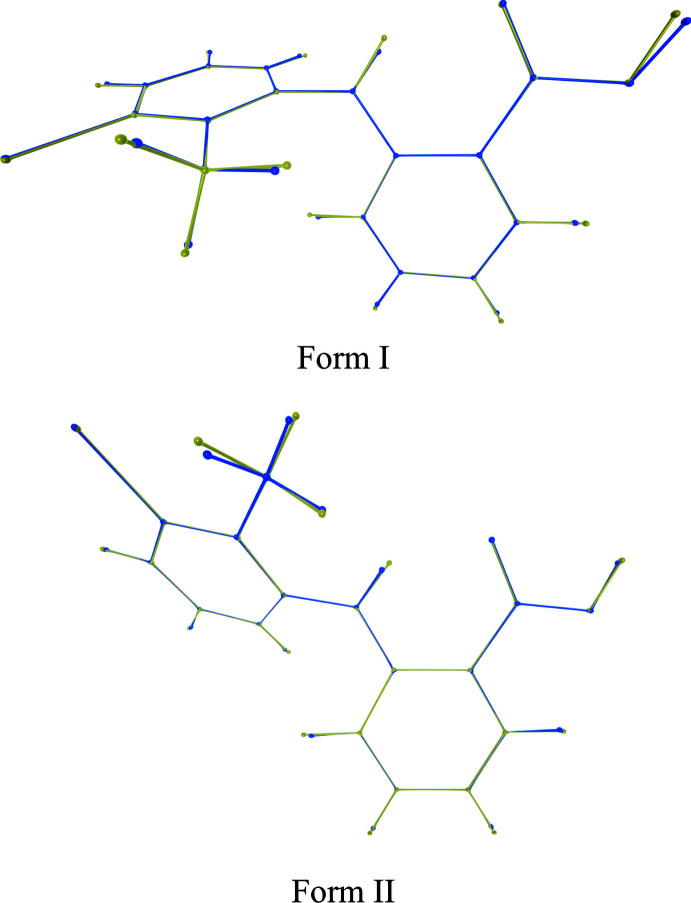
Overlay of asymmetric units of form I and form II, comparing the positions of hydrogen atoms in this work (orange) to previous work (blue; CSD refcodes form I: KAXXAI01, form II: KAXXAI; Andersen *et al.*, 1989 ▸). Figure produced using *4Sight* (C4X Discovery, UK).

The database structures with refcodes KAXXAI01 and KAXXAI correspond to the crystal structures of form I and form II redetermined here at higher resolution. The locations of the heavy atoms in these new structures do not differ significantly from those reported previously even though, as expected, some hydrogen-atom locations differ substanti­ally (Fig. 5 ▸). H7 is in a significantly different position in both structures, materially affecting both its associated hydrogen-bond geometries (compare Tables 1 ▸ and 2 ▸ with Table 3 ▸) and covalent bond lengths. The N—H bond length is some 23% longer for form I and 17% for form II, which would correspond to a considerable calculated difference in residual dipolar couplings by factors of 1.9 and 1.6 times smaller, respectively. The O—H bond length is slightly longer by 5% for form I and 6% for form II. Carbon–hydrogen bond lengths are also notably longer at 13 ± 3% (min. 6%, max. 17%) for form I and 10 ± 3% (min. 5%, max. 14%) for form II; C—C—H bond angles differ absolutely by 2.6 ± 1.8% (min. 0.0, max 5.7°) for form I and 1.6 ± 1.3° (min. 0.2, max 5.2°) for form II. This improved precision in hydrogen-atom placement provides a significant structural enhancement for subsequent solid- and solution-state NMR studies.

## Synthesis and crystallization   

Tolfenamic acid was used as received from Sigma–Aldrich (Gillingham, UK).

Large single crystals of form I (needles 0.5–3 mm in length) were grown by slow evaporation at room temperature: 3 mg of compound was dissolved in an initial volume of 200 µl of ethyl acetate and the mixture was allowed to evaporate to dryness over 24–36 h.

Large single crystals of form II (needles 0.3–1 mm in length) were obtained serendipitously from an attempted salt crystallization experiment, during which form II crystals suitable for single-crystal X-ray diffraction were isolated. A 1:1 molar ratio of TFA (20 mg of compound) and *N*-(2-hy­droxy­eth­yl)pyrrolidine were dissolved into approximately 5 ml ethyl acetate. The mixture was then left to slowly evaporate, during which large yellow needles formed. Single-crystal diffraction performed on multiple crystals indicated that these were pure form II. There was no evidence of form I within the product, nor of any inclusion of *N*-(2-hy­droxy­eth­yl)-pyrrolidine within the form II crystals. Clearly the presence of *N*-(2-hy­droxy­eth­yl)-pyrrolidine either inhibited the growth of form I crystals and/or promoted the growth of form II crystals; the mechanism for this has not been investigated.

## Refinement   

Crystal data, data collection and structure refinement details are summarized in Table 4 ▸.

Intensity data for form I and form II were collected using Mo *K*α radiation at 100 and 150 K respectively using a Rigaku FR-X rotating anode diffractometer, equipped with an HyPix-6000HE detector and an Oxford Cryosystems nitro­gen flow gas system. Data were measured and reduced using the *CrysAlis PRO* suite of programs. Absorption correction was performed using empirical methods implemented in the SCALE3 ABSPACK scaling algorithm (Blessing, 1995 ▸; Sheldrick, 1996 ▸). The crystal structures were solved and refined against all *F* ^2^ values using the *SHELXL* and *OLEX2* suite of programs (Sheldrick, 2008 ▸, 2015*b* ▸; Dolomanov *et al.*, 2009 ▸). All atoms (including H atoms) were refined anisotropically.

Hirshfeld atom refinement was achieved using the recently implemented HARt option in *OLEX2* (Fugel *et al.*, 2018 ▸). It precisely estimates the atomic positions and deformation electron densities in crystal structures by deconvolution of accurate static electron density calculated by *TONTO* from the thermally smeared electron density calculated from an independent atomic model (IAM) obtained from the X-ray diffraction data (Capelli *et al.*, 2014 ▸).

The wavefunctions of crystal structures of form I and form II were calculated using *TONTO* (Capelli *et al.*, 2014 ▸), with the cc-pVTZ basis set (Dunning, 1989 ▸) and the Hartree–Fock method. Wavefunctions for each crystal structure were calculated with the crystal structure grown in order to account for the hydrogen bond formed between the carboxyl­ate groups. Each model obtained was then refined against the single crystal X-ray diffraction data collected, using *OLEX2* and the L-M method (Fugel *et al.*, 2018 ▸).

## Supplementary Material

Crystal structure: contains datablock(s) global, I, II. DOI: 10.1107/S2056989020010841/hb7936sup1.cif

Supporting information file. DOI: 10.1107/S2056989020010841/hb7936Isup2.cml

CCDC references: 1960855, 1960856

Additional supporting information:  crystallographic information; 3D view; checkCIF report

## Figures and Tables

**Figure 4:**
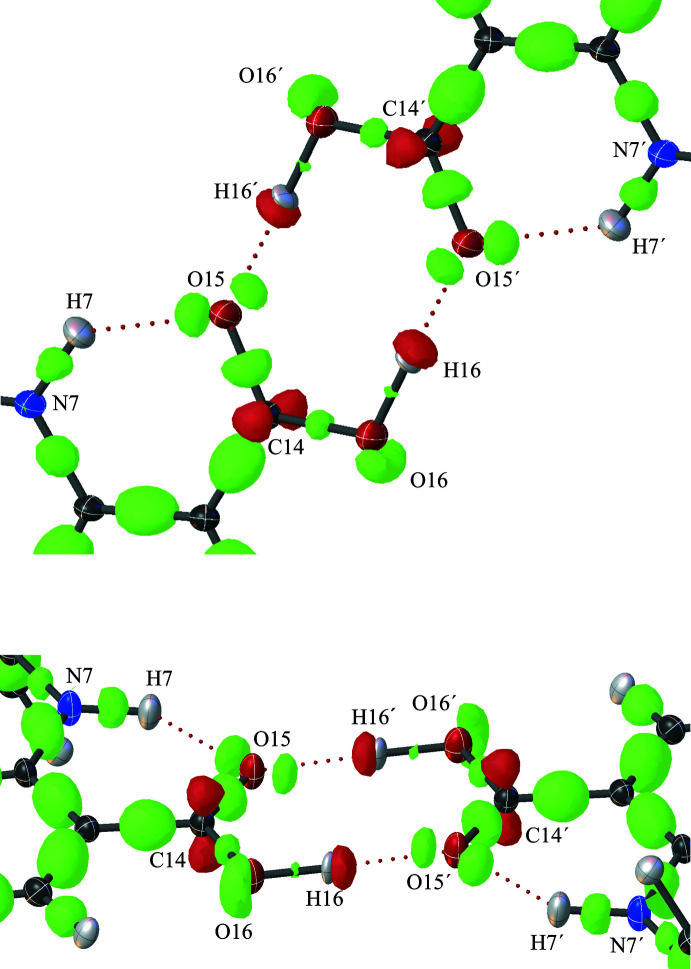
Detail of the hydrogen-bond network of TFA form I in two orientations, showing the hydrogen bonds (red dotted lines) and the deformation of the electron density calculated by *TONTO* (Capelli *et al.*, 2014 ▸). The differing mol­ecular orbitals for O15 and O16 are clearly visible. Negative electron density is shown with green surfaces and positive electron density is shown with red (threshold level −0.25, Res/Å). Ellipsoids of carbon atoms are shown in grey, nitro­gen blue, oxygen red and hydrogen white.

**Table 1:** Hydrogen-bond geometry (Å, °) for form I[Chem scheme1]

*D*—H⋯*A*	*D*—H	H⋯*A*	*D*⋯*A*	*D*—H⋯*A*
N7—H7⋯O15	1.02 (2)	1.85 (2)	2.6650 (10)	134.0 (19)
O16—H16⋯O15^i^	1.02 (2)	1.63 (2)	2.6448 (10)	175 (2)
C18—H18*A*⋯Cl17^ii^	1.09 (3)	2.81 (3)	3.8674 (11)	163 (2)

Symmetry codes: (i) 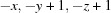; (ii) 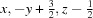.

**Table 2:** Hydrogen-bond geometry (Å, °) for form II[Chem scheme1]

*D*—H⋯*A*	*D*—H	H⋯*A*	*D*⋯*A*	*D*—H⋯*A*
N7—H7⋯O15	1.019 (11)	1.800 (10)	2.6469 (7)	138.0 (8)
O16—H16⋯O15^i^	0.998 (14)	1.640 (14)	2.6381 (8)	179.0 (12)

Symmetry code: (i) 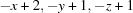.

**Table 3:** Hydrogen-bond geometry (Å, °) for structures of forms I and II determined by Andersen *et al.* (1989 ▸)

Form	*D*—H⋯*A*	*D*—H	H⋯*A*	*D*⋯*A*	*D*—H⋯*A*
I (KAXXAI01)	N7—H7⋯O15	0.79	2.02	2.676	141
	O16—H16⋯O15^i^	0.97	1.69	2.648	170
					
II (KAXXAI)	N7—H7⋯O15	0.84	1.96	2.653	139
	O16—H16⋯O15^ii^	0.93	1.72	2.648	176

Symmetry codes: (i) −*x*, 1 − *y*, 1 − *z*; (ii) 2 − *x*, 1 − *y*, 1 − *z*.

**Table 4:** Experimental details

	form I	form II
Crystal data		
Chemical formula	C_14_H_12_ClNO_2_	C_14_H_12_ClNO_2_
*M*_r_	261.71	261.71
Crystal system, space group	Monoclinic, *P*2_1_/*c*	Monoclinic, *P*2_1_/*n*
Temperature (K)	100	150
*a*, *b*, *c* (Å)	4.8283 (2), 32.0832 (10), 8.0221 (3)	3.84618 (14), 21.9502 (7), 14.1764 (5)
β (°)	104.936 (4)	94.235 (4)
*V* (Å^3^)	1200.70 (8)	1193.57 (7)
*Z*	4	4
Radiation type	Mo *K*α	Mo *K*α
μ (mm^−1^)	0.31	0.31
Crystal size (mm)	1.0 × 0.4 × 0.2	0.55 × 0.05 × 0.05

Data collection		
Diffractometer	XtaLAB AFC11 (RINC): Kappa single	XtaLAB AFC11 (RINC): Kappa single
Absorption correction	Multi-scan (*CrysAlis PRO*; Rigaku, 2017 ▸)	Multi-scan (*CrysAlis PRO*; Rigaku, 2017 ▸)
*T*_min_, *T*_max_	0.457, 1.000	0.154, 1.000
No. of measured, independent and observed [*I* ≥ 2σ(*I*)] reflections	39154, 11328, 8616	22189, 7967, 5973
*R*_int_	0.039	0.032
(sin θ/λ)_max_ (Å^−1^)	1.042	0.938

Refinement		
*R*[*F* ^2^ > 2σ(*F* ^2^)], *wR*(*F* ^2^), *S*	0.064, 0.127, 1.04	0.046, 0.077, 1.10
No. of reflections	11328	7967
No. of parameters	271	271
H-atom treatment	All H-atom parameters refined	All H-atom parameters refined
Δρ_max_, Δρ_min_ (e Å^−3^)	0.82, −0.69	0.81, −0.54

Computer programs: *CrysAlis PRO* (Rigaku OD, 2017 ▸), *SHELXT* (Sheldrick, 2015*a* ▸), *olex2.refine* (Bourhis *et al.*, 2015 ▸) and *OLEX2* (Dolomanov *et al.*, 2009 ▸).

## supplementary crystallographic information

### 2-(3-Chloro-2-methylanilino)benzoic acid (I) Crystal data

**Table:**

C_14_H_12_ClNO_2_	*F*(000) = 544.838
*M**_r_* = 261.71	*D*_x_ = 1.448 Mg m^−^^3^
Monoclinic, *P*2_1_/*c*	Mo *K*α radiation, λ = 0.71073 Å
*a* = 4.8283 (2) Å	Cell parameters from 9015 reflections
*b* = 32.0832 (10) Å	θ = 3.8–52.9°
*c* = 8.0221 (3) Å	µ = 0.31 mm^−^^1^
β = 104.936 (4)°	*T* = 100 K
*V* = 1200.70 (8) Å^3^	Plank, clear light colourless
*Z* = 4	1.0 × 0.4 × 0.2 mm

### 2-(3-Chloro-2-methylanilino)benzoic acid (I) Data collection

**Table:**

XtaLAB AFC11 (RINC): Kappa single diffractometer	11328 independent reflections
Radiation source: fine-focus sealed X-ray tube, Enhance (Mo) X-ray Source	8616 reflections with *I*≥ 2σ(*I*)
Graphite monochromator	*R*_int_ = 0.039
ω scans	θ_max_ = 47.8°, θ_min_ = 3.8°
Absorption correction: multi-scan (CrysAlisPro; Rigaku, 2017)	*h* = −10→6
*T*_min_ = 0.457, *T*_max_ = 1.000	*k* = −62→72
39154 measured reflections	*l* = −18→18

### 2-(3-Chloro-2-methylanilino)benzoic acid (I) Refinement

**Table:**

Refinement on *F*^2^	0 constraints
Least-squares matrix: full	Primary atom site location: dual
*R*[*F*^2^ > 2σ(*F*^2^)] = 0.064	All H-atom parameters refined
*wR*(*F*^2^) = 0.127	*w* = 1/[σ^2^(*F*_o_^2^) + (0.*P*)^2^ + 0.987*P*] where *P* = (*F*_o_^2^ + 2*F*_c_^2^)/3
*S* = 1.04	(Δ/σ)_max_ = 0.0004
11328 reflections	Δρ_max_ = 0.82 e Å^−^^3^
271 parameters	Δρ_min_ = −0.69 e Å^−^^3^
0 restraints	

### 2-(3-Chloro-2-methylanilino)benzoic acid (I) Special details

**Table:**

Refinement. Refinement using NoSpherA2, an implementation of NOn-SPHERical Atom-form-factors in Olex2. Please cite:F. Kleemiss, H. Puschmann, O. Dolomanov, S.Grabowsky - to be publsihed - 2020 NoSpherA2 makes use of tailor-made aspherical atomic form factors calculated on-the-fly from a Hirshfeld-partitioned electron density (ED) - not from spherical-atom form factors.The ED is calculated from a gaussian basis set single determinant SCF wavefunction - either Hartree-Fock or B3LYP - for a fragment of the crystal embedded in an electrostatic crystal field. The following options were used: SOFTWARE: Tonto METHOD: rhf BASIS SET: def2-SVP CHARGE: 0 MULTIPLICITY: 1 DATE: 2019-10-18_17-21-04 CLUSTER RADIUS: 0

### 2-(3-Chloro-2-methylanilino)benzoic acid (I) Fractional atomic coordinates and isotropic or equivalent isotropic displacement parameters (Å^2^)

**Table:**

	*x*	*y*	*z*	*U*_iso_*/*U*_eq_	
Cl17	0.75639 (6)	0.759893 (7)	0.58628 (4)	0.02423 (5)	
O16	0.07003 (16)	0.49986 (2)	0.28719 (9)	0.01785 (11)	
O15	0.23425 (15)	0.53749 (2)	0.52534 (9)	0.01694 (11)	
N7	0.58438 (18)	0.60116 (2)	0.51594 (10)	0.01698 (12)	
C9	0.39509 (16)	0.55388 (2)	0.27629 (10)	0.01206 (10)	
C8	0.56974 (17)	0.58797 (2)	0.35212 (10)	0.01279 (11)	
C10	0.38168 (19)	0.54232 (3)	0.10596 (11)	0.01506 (12)	
C6	0.66873 (18)	0.67645 (3)	0.54885 (11)	0.01456 (12)	
C5	0.84386 (19)	0.70814 (3)	0.63887 (12)	0.01590 (13)	
C14	0.22811 (17)	0.53019 (2)	0.37277 (11)	0.01293 (11)	
C13	0.73019 (19)	0.60848 (3)	0.25247 (12)	0.01624 (13)	
C11	0.5414 (2)	0.56279 (3)	0.01027 (12)	0.01743 (14)	
C1	0.75379 (18)	0.63527 (3)	0.59655 (11)	0.01417 (11)	
C2	1.0000 (2)	0.62691 (3)	0.72745 (12)	0.01740 (13)	
C3	1.1660 (2)	0.65937 (3)	0.81486 (13)	0.02070 (16)	
C4	1.0886 (2)	0.70036 (3)	0.77028 (13)	0.01962 (15)	
C12	0.7174 (2)	0.59587 (3)	0.08645 (12)	0.01759 (14)	
C18	0.4031 (2)	0.68604 (3)	0.41021 (15)	0.02055 (16)	
H10	0.245 (4)	0.5165 (5)	0.050 (2)	0.032 (4)	
H12	0.851 (4)	0.6117 (6)	0.015 (3)	0.039 (5)	
H2	1.055 (4)	0.5947 (6)	0.760 (3)	0.035 (5)	
H4	1.216 (4)	0.7266 (6)	0.835 (3)	0.040 (5)	
H3	1.356 (4)	0.6530 (7)	0.919 (3)	0.048 (6)	
H13	0.868 (4)	0.6342 (6)	0.312 (2)	0.039 (5)	
H11	0.535 (4)	0.5528 (6)	−0.120 (2)	0.040 (5)	
H16	−0.038 (5)	0.4851 (7)	0.364 (3)	0.028 (6)	
H7	0.484 (5)	0.5821 (7)	0.584 (3)	0.045 (6)	
H18a	0.455 (5)	0.7026 (9)	0.303 (3)	0.071 (8)	
H18b	0.291 (5)	0.6586 (7)	0.363 (4)	0.078 (9)	
H18c	0.264 (5)	0.7068 (8)	0.451 (3)	0.067 (8)	

### 2-(3-Chloro-2-methylanilino)benzoic acid (I) Atomic displacement parameters (Å^2^)

**Table:**

	*U*^11^	*U*^22^	*U*^33^	*U*^12^	*U*^13^	*U*^23^
Cl17	0.03142 (12)	0.01403 (8)	0.02626 (12)	−0.00050 (8)	0.00568 (9)	0.00054 (8)
O16	0.0216 (3)	0.0181 (3)	0.0145 (2)	−0.0072 (2)	0.0059 (2)	−0.0029 (2)
O15	0.0214 (3)	0.0177 (3)	0.0128 (2)	−0.0073 (2)	0.0065 (2)	−0.00110 (19)
N7	0.0226 (3)	0.0172 (3)	0.0131 (3)	−0.0077 (2)	0.0082 (2)	−0.0018 (2)
C9	0.0133 (3)	0.0127 (2)	0.0106 (2)	−0.0002 (2)	0.0040 (2)	0.0006 (2)
C8	0.0140 (3)	0.0142 (3)	0.0115 (3)	−0.0020 (2)	0.0057 (2)	0.0005 (2)
C10	0.0181 (3)	0.0162 (3)	0.0116 (3)	0.0000 (2)	0.0051 (2)	−0.0005 (2)
C6	0.0137 (3)	0.0153 (3)	0.0150 (3)	−0.0024 (2)	0.0042 (2)	0.0002 (2)
C5	0.0176 (3)	0.0148 (3)	0.0157 (3)	−0.0022 (2)	0.0048 (2)	−0.0015 (2)
C14	0.0144 (3)	0.0127 (3)	0.0122 (3)	−0.0019 (2)	0.0043 (2)	0.0000 (2)
C13	0.0180 (3)	0.0186 (3)	0.0141 (3)	−0.0037 (2)	0.0079 (2)	0.0010 (2)
C11	0.0226 (4)	0.0193 (3)	0.0120 (3)	0.0012 (3)	0.0074 (3)	0.0014 (2)
C1	0.0164 (3)	0.0147 (3)	0.0123 (3)	−0.0032 (2)	0.0052 (2)	−0.0005 (2)
C2	0.0203 (3)	0.0163 (3)	0.0148 (3)	0.0002 (3)	0.0031 (3)	0.0004 (2)
C3	0.0212 (4)	0.0206 (4)	0.0171 (3)	−0.0003 (3)	−0.0009 (3)	−0.0018 (3)
C4	0.0206 (4)	0.0184 (3)	0.0180 (3)	−0.0037 (3)	0.0014 (3)	−0.0028 (3)
C12	0.0202 (3)	0.0207 (3)	0.0145 (3)	−0.0007 (3)	0.0092 (3)	0.0022 (3)
C18	0.0150 (3)	0.0231 (4)	0.0221 (4)	−0.0005 (3)	0.0022 (3)	0.0012 (3)
H10	0.045 (12)	0.030 (10)	0.020 (10)	−0.008 (9)	0.006 (9)	0.002 (8)
H12	0.045 (12)	0.041 (12)	0.040 (13)	−0.013 (10)	0.025 (11)	−0.003 (10)
H2	0.039 (12)	0.032 (11)	0.035 (12)	−0.002 (9)	0.009 (10)	0.001 (9)
H4	0.047 (13)	0.039 (12)	0.033 (13)	−0.009 (10)	0.009 (10)	0.003 (10)
H3	0.033 (12)	0.071 (16)	0.031 (13)	0.000 (11)	−0.007 (10)	0.002 (11)
H13	0.053 (13)	0.043 (12)	0.024 (11)	−0.015 (10)	0.015 (10)	0.001 (9)
H11	0.048 (13)	0.042 (12)	0.030 (12)	−0.006 (10)	0.010 (10)	−0.010 (10)
H16	0.029 (13)	0.029 (13)	0.028 (14)	−0.018 (11)	0.010 (11)	0.001 (11)
H7	0.059 (17)	0.039 (14)	0.044 (15)	−0.019 (12)	0.026 (13)	−0.001 (12)
H18a	0.043 (15)	0.11 (2)	0.048 (16)	0.000 (15)	0.001 (12)	0.031 (16)
H18b	0.038 (13)	0.039 (13)	0.12 (2)	−0.007 (11)	−0.035 (14)	0.014 (15)
H18c	0.047 (15)	0.09 (2)	0.061 (18)	0.032 (14)	0.007 (13)	0.006 (15)

### 2-(3-Chloro-2-methylanilino)benzoic acid (I) Geometric parameters (Å, º)

**Table:**

Cl17—C5	1.7383 (9)	C5—C4	1.3887 (13)
O16—C14	1.3154 (10)	C13—C12	1.3780 (13)
O16—H16	1.02 (2)	C13—H13	1.090 (19)
O15—C14	1.2389 (11)	C11—C12	1.3979 (14)
N7—C8	1.3650 (11)	C11—H11	1.087 (19)
N7—C1	1.4184 (11)	C1—C2	1.3943 (13)
N7—H7	1.02 (2)	C2—C3	1.3890 (14)
C9—C8	1.4188 (11)	C2—H2	1.081 (18)
C9—C10	1.4012 (11)	C3—C4	1.3885 (14)
C9—C14	1.4659 (11)	C3—H3	1.089 (19)
C8—C13	1.4110 (11)	C4—H4	1.093 (19)
C10—C11	1.3858 (12)	C12—H12	1.092 (17)
C10—H10	1.080 (17)	C18—H18a	1.09 (2)
C6—C5	1.3983 (12)	C18—H18b	1.05 (2)
C6—C1	1.4072 (12)	C18—H18c	1.06 (2)
C6—C18	1.4969 (13)		
			
H16—O16—C14	110.3 (12)	C12—C11—C10	118.69 (8)
C1—N7—C8	123.97 (7)	H11—C11—C10	120.9 (10)
H7—N7—C8	114.6 (13)	H11—C11—C12	120.4 (10)
H7—N7—C1	120.9 (13)	C6—C1—N7	120.45 (8)
C10—C9—C8	119.68 (7)	C2—C1—N7	118.33 (8)
C14—C9—C8	121.36 (7)	C2—C1—C6	121.18 (8)
C14—C9—C10	118.95 (7)	C3—C2—C1	120.34 (9)
C9—C8—N7	121.96 (7)	H2—C2—C1	118.3 (10)
C13—C8—N7	120.08 (8)	H2—C2—C3	121.3 (10)
C13—C8—C9	117.96 (7)	C4—C3—C2	119.87 (9)
C11—C10—C9	121.48 (8)	H3—C3—C2	120.6 (12)
H10—C10—C9	118.4 (10)	H3—C3—C4	119.5 (12)
H10—C10—C11	120.2 (10)	C3—C4—C5	119.05 (9)
C1—C6—C5	116.55 (8)	H4—C4—C5	119.1 (11)
C18—C6—C5	121.50 (8)	H4—C4—C3	121.8 (11)
C18—C6—C1	121.95 (8)	C11—C12—C13	121.07 (8)
C6—C5—Cl17	119.50 (7)	H12—C12—C13	119.0 (10)
C4—C5—Cl17	117.51 (7)	H12—C12—C11	119.9 (10)
C4—C5—C6	122.99 (8)	H18a—C18—C6	111.1 (12)
O15—C14—O16	121.23 (7)	H18b—C18—C6	111.0 (12)
C9—C14—O16	115.60 (7)	H18b—C18—H18a	109 (2)
C9—C14—O15	123.17 (7)	H18c—C18—C6	113.1 (13)
C12—C13—C8	121.09 (8)	H18c—C18—H18a	103 (2)
H13—C13—C8	117.8 (10)	H18c—C18—H18b	109 (2)
H13—C13—C12	121.1 (10)		
			
Cl17—C5—C6—C1	179.26 (7)	N7—C1—C6—C18	−1.38 (10)
Cl17—C5—C6—C18	−1.32 (9)	N7—C1—C2—C3	−177.36 (9)
Cl17—C5—C4—C3	−179.56 (8)	C9—C8—C13—C12	0.13 (9)
O16—C14—C9—C8	178.63 (7)	C9—C10—C11—C12	−0.45 (10)
O16—C14—C9—C10	−1.64 (9)	C8—C13—C12—C11	1.09 (11)
O15—C14—C9—C8	−1.98 (10)	C10—C11—C12—C13	−0.93 (11)
O15—C14—C9—C10	177.76 (8)	C6—C5—C4—C3	0.35 (11)
N7—C8—C9—C10	178.26 (8)	C6—C1—C2—C3	0.52 (10)
N7—C8—C9—C14	−2.01 (10)	C5—C4—C3—C2	0.40 (12)
N7—C8—C13—C12	−179.60 (9)	C1—C2—C3—C4	−0.82 (11)
N7—C1—C6—C5	178.03 (8)		

### 2-(3-Chloro-2-methylanilino)benzoic acid (I) Hydrogen-bond geometry (Å, º)

**Table:**

*D*—H···*A*	*D*—H	H···*A*	*D*···*A*	*D*—H···*A*
N7—H7···O15	1.02 (2)	1.85 (2)	2.6650 (10)	134.0 (19)
O16—H16···O15^i^	1.02 (2)	1.63 (2)	2.6448 (10)	175 (2)
C18—H18A···Cl17^ii^	1.09 (3)	2.81 (3)	3.8674 (11)	163 (2)

Symmetry codes: (i) −*x*, −*y*+1, −*z*+1; (ii) *x*, −*y*+3/2, *z*−1/2.

### (II) Crystal data

**Table:**

C_14_H_12_ClNO_2_	*F*(000) = 544.838
*M**_r_* = 261.71	*D*_x_ = 1.456 Mg m^−^^3^
Monoclinic, *P*2_1_/*n*	Mo *K*α radiation, λ = 0.71073 Å
*a* = 3.84618 (14) Å	Cell parameters from 6709 reflections
*b* = 21.9502 (7) Å	θ = 2.3–40.6°
*c* = 14.1764 (5) Å	µ = 0.31 mm^−^^1^
β = 94.235 (4)°	*T* = 150 K
*V* = 1193.57 (7) Å^3^	Needle, clear light colourless
*Z* = 4	0.55 × 0.05 × 0.05 mm

### (II) Data collection

**Table:**

XtaLAB AFC11 (RINC): Kappa single diffractometer	7967 independent reflections
Radiation source: fine-focus sealed X-ray tube, Enhance (Mo) X-ray Source	5973 reflections with *I*≥ 2σ(*I*)
Graphite monochromator	*R*_int_ = 0.032
ω scans	θ_max_ = 41.8°, θ_min_ = 2.4°
Absorption correction: multi-scan (CrysAlisPro; Rigaku, 2017)	*h* = −7→7
*T*_min_ = 0.154, *T*_max_ = 1.000	*k* = −33→40
22189 measured reflections	*l* = −26→19

### (II) Refinement

**Table:**

Refinement on *F*^2^	0 constraints
Least-squares matrix: full	Primary atom site location: dual
*R*[*F*^2^ > 2σ(*F*^2^)] = 0.046	All H-atom parameters refined
*wR*(*F*^2^) = 0.077	*w* = 1/[σ^2^(*F*_o_^2^) + (0.0284*P*)^2^] where *P* = (*F*_o_^2^ + 2*F*_c_^2^)/3
*S* = 1.10	(Δ/σ)_max_ = 0.001
7967 reflections	Δρ_max_ = 0.81 e Å^−^^3^
271 parameters	Δρ_min_ = −0.54 e Å^−^^3^
0 restraints	

### (II) Special details

**Table:**

Refinement. Refinement using NoSpherA2, an implementation of NOn-SPHERical A tom-form-factors in Olex2. Please cite:F. Kleemiss, H. Puschmann, O. Dolomanov, S.Grabowsky - to be publsihed - 2020 NoSpherA2 makes use of tailor-made aspherical atomic form factors calculated on-the-fly from a Hirshfeld-partitioned electron density (ED) - not from spherical-atom form factors.The ED is calculated from a gaussian basis set single determinant SCF wavefunction - either Hartree-Fock or B3LYP - for a fragment of the crystal embedded in an electrostatic crystal field. The following options were used: SOFTWARE: Tonto METHOD: rhf BASIS SET: def2-SVP CHARGE: 0 MULTIPLICITY: 1 DATE: 2019-10-22_21-03-05 CLUSTER RADIUS: 0

### (II) Fractional atomic coordinates and isotropic or equivalent isotropic displacement parameters (Å^2^)

**Table:**

	*x*	*y*	*z*	*U*_iso_*/*U*_eq_	
Cl17	0.16061 (4)	0.163247 (7)	0.476783 (12)	0.02253 (4)	
C5	0.30783 (14)	0.20431 (2)	0.38283 (4)	0.01663 (10)	
C6	0.34927 (14)	0.26716 (2)	0.39149 (4)	0.01586 (10)	
C8	0.48962 (13)	0.40676 (2)	0.26010 (4)	0.01554 (10)	
C2	0.55157 (16)	0.26655 (3)	0.23271 (5)	0.01887 (11)	
C1	0.47434 (14)	0.29837 (2)	0.31392 (4)	0.01676 (10)	
C4	0.37873 (15)	0.17219 (3)	0.30233 (5)	0.01955 (11)	
C9	0.60895 (14)	0.46663 (2)	0.28302 (4)	0.01549 (10)	
O16	0.89072 (14)	0.53791 (2)	0.38625 (4)	0.02738 (11)	
C11	0.39417 (16)	0.50278 (3)	0.12791 (5)	0.02097 (12)	
N7	0.54116 (16)	0.36075 (2)	0.32481 (4)	0.02311 (11)	
C13	0.31026 (14)	0.39744 (2)	0.17112 (4)	0.01695 (10)	
O15	0.81422 (14)	0.44483 (2)	0.44188 (4)	0.02923 (12)	
C10	0.56103 (15)	0.51329 (2)	0.21575 (5)	0.01865 (11)	
C3	0.50114 (16)	0.20406 (3)	0.22700 (5)	0.02053 (11)	
C12	0.26535 (15)	0.44447 (3)	0.10676 (5)	0.01917 (11)	
C14	0.77823 (15)	0.48161 (2)	0.37589 (5)	0.01896 (11)	
C18	0.26524 (18)	0.29999 (3)	0.47953 (5)	0.02301 (12)	
H13	0.202 (2)	0.3529 (4)	0.1536 (7)	0.036 (2)	
H10	0.657 (3)	0.5586 (4)	0.2342 (7)	0.042 (3)	
H2	0.662 (2)	0.2909 (4)	0.1761 (7)	0.039 (2)	
H4	0.340 (3)	0.1233 (4)	0.3007 (8)	0.046 (3)	
H11	0.358 (3)	0.5389 (5)	0.0775 (9)	0.055 (3)	
H12	0.121 (3)	0.4359 (4)	0.0404 (8)	0.043 (3)	
H3	0.560 (3)	0.1790 (5)	0.1640 (8)	0.045 (3)	
H7	0.635 (3)	0.3758 (4)	0.3898 (8)	0.042 (3)	
H16	1.001 (4)	0.5449 (6)	0.4513 (10)	0.039 (3)	
H18A	0.043 (3)	0.2800 (6)	0.5101 (10)	0.070 (4)	
H18B	0.199 (5)	0.3458 (5)	0.4672 (10)	0.093 (6)	
H18C	0.466 (3)	0.2985 (8)	0.5334 (9)	0.089 (5)	

### (II) Atomic displacement parameters (Å^2^)

**Table:**

	*U*^11^	*U*^22^	*U*^33^	*U*^12^	*U*^13^	*U*^23^
Cl17	0.02704 (7)	0.01987 (6)	0.02118 (8)	−0.00156 (5)	0.00511 (5)	0.00733 (5)
C5	0.0188 (2)	0.0144 (2)	0.0168 (3)	−0.00081 (16)	0.00133 (19)	0.00219 (18)
C6	0.0194 (2)	0.0147 (2)	0.0134 (2)	−0.00062 (16)	0.00050 (19)	0.00098 (18)
C8	0.0197 (2)	0.0135 (2)	0.0132 (2)	−0.00254 (16)	−0.00056 (18)	−0.00060 (17)
C2	0.0249 (3)	0.0176 (2)	0.0144 (3)	−0.00347 (19)	0.0033 (2)	0.0002 (2)
C1	0.0226 (2)	0.0138 (2)	0.0137 (3)	−0.00380 (17)	−0.00006 (19)	0.00040 (18)
C4	0.0251 (3)	0.0138 (2)	0.0199 (3)	−0.00124 (17)	0.0027 (2)	0.00004 (19)
C9	0.0187 (2)	0.0128 (2)	0.0146 (2)	−0.00135 (16)	−0.00103 (19)	−0.00116 (17)
O16	0.0437 (3)	0.01627 (18)	0.0208 (2)	−0.00765 (17)	−0.0076 (2)	−0.00184 (17)
C11	0.0278 (3)	0.0165 (2)	0.0179 (3)	0.00172 (19)	−0.0031 (2)	0.0022 (2)
N7	0.0388 (3)	0.0147 (2)	0.0149 (2)	−0.00749 (18)	−0.0045 (2)	0.00098 (17)
C13	0.0196 (2)	0.0160 (2)	0.0148 (3)	−0.00141 (17)	−0.00178 (19)	−0.00154 (18)
O15	0.0505 (3)	0.01728 (19)	0.0180 (2)	−0.00828 (18)	−0.0106 (2)	−0.00045 (16)
C10	0.0240 (2)	0.0132 (2)	0.0182 (3)	−0.00090 (18)	−0.0019 (2)	0.00082 (19)
C3	0.0279 (3)	0.0167 (2)	0.0172 (3)	−0.00086 (19)	0.0038 (2)	−0.0019 (2)
C12	0.0222 (2)	0.0184 (2)	0.0162 (3)	0.00175 (18)	−0.0037 (2)	−0.0005 (2)
C14	0.0264 (3)	0.0137 (2)	0.0161 (3)	−0.00261 (17)	−0.0035 (2)	−0.00178 (19)
C18	0.0300 (3)	0.0226 (3)	0.0166 (3)	0.0012 (2)	0.0029 (2)	−0.0015 (2)
H13	0.040 (6)	0.040 (5)	0.027 (7)	−0.011 (5)	−0.002 (5)	−0.012 (5)
H10	0.051 (6)	0.032 (6)	0.041 (7)	−0.007 (5)	−0.005 (5)	0.004 (5)
H2	0.046 (6)	0.038 (6)	0.033 (7)	−0.004 (5)	0.010 (5)	−0.002 (5)
H4	0.070 (7)	0.020 (5)	0.052 (8)	−0.002 (5)	0.025 (6)	−0.003 (5)
H11	0.074 (8)	0.033 (6)	0.057 (9)	0.008 (5)	−0.008 (6)	0.006 (6)
H12	0.052 (6)	0.033 (6)	0.041 (7)	−0.001 (4)	−0.013 (5)	0.004 (5)
H3	0.064 (7)	0.031 (6)	0.040 (8)	−0.001 (5)	−0.002 (6)	−0.007 (5)
H7	0.069 (9)	0.023 (6)	0.032 (8)	−0.010 (5)	−0.003 (6)	−0.008 (5)
H16	0.053 (8)	0.037 (7)	0.024 (8)	−0.002 (6)	−0.007 (7)	−0.002 (6)
H18A	0.063 (8)	0.080 (9)	0.071 (11)	−0.015 (7)	0.036 (7)	−0.023 (8)
H18B	0.201 (18)	0.029 (7)	0.051 (11)	0.024 (8)	0.022 (11)	−0.008 (6)
H18C	0.070 (9)	0.145 (14)	0.051 (10)	0.048 (9)	−0.014 (7)	−0.054 (9)

### (II) Geometric parameters (Å, º)

**Table:**

Cl17—C5	1.7373 (6)	O16—C14	1.3136 (7)
C5—C6	1.3933 (8)	O16—H16	0.983 (14)
C5—C4	1.3850 (9)	C11—C10	1.3761 (9)
C6—C1	1.4103 (9)	C11—C12	1.3957 (9)
C6—C18	1.4957 (9)	C11—H11	1.085 (10)
C8—C9	1.4217 (7)	N7—H7	1.019 (10)
C8—N7	1.3679 (8)	C13—C12	1.3789 (8)
C8—C13	1.4064 (8)	C13—H13	1.089 (9)
C2—C1	1.3965 (9)	O15—C14	1.2355 (8)
C2—C3	1.3855 (9)	C10—H10	1.088 (9)
C2—H2	1.081 (10)	C3—H3	1.098 (10)
C1—N7	1.3985 (7)	C12—H12	1.078 (10)
C4—C3	1.3865 (10)	C18—H18A	1.087 (12)
C4—H4	1.086 (9)	C18—H18B	1.048 (11)
C9—C10	1.4019 (8)	C18—H18C	1.052 (11)
C9—C14	1.4625 (8)		
			
C6—C5—Cl17	119.20 (5)	C1—N7—C8	129.34 (5)
C4—C5—Cl17	117.59 (4)	H7—N7—C8	113.1 (7)
C4—C5—C6	123.21 (6)	H7—N7—C1	117.5 (7)
C1—C6—C5	117.11 (6)	C12—C13—C8	120.91 (5)
C18—C6—C5	121.33 (6)	H13—C13—C8	119.5 (6)
C18—C6—C1	121.55 (5)	H13—C13—C12	119.6 (6)
N7—C8—C9	120.11 (5)	C11—C10—C9	121.44 (5)
C13—C8—C9	117.85 (5)	H10—C10—C9	118.6 (6)
C13—C8—N7	122.00 (5)	H10—C10—C11	119.9 (6)
C3—C2—C1	120.37 (6)	C4—C3—C2	120.55 (6)
H2—C2—C1	118.9 (6)	H3—C3—C2	120.6 (6)
H2—C2—C3	120.7 (6)	H3—C3—C4	118.8 (6)
C2—C1—C6	120.31 (5)	C13—C12—C11	121.20 (6)
N7—C1—C6	117.43 (6)	H12—C12—C11	120.0 (5)
N7—C1—C2	122.09 (6)	H12—C12—C13	118.7 (5)
C3—C4—C5	118.43 (5)	O16—C14—C9	115.61 (5)
H4—C4—C5	119.2 (6)	O15—C14—C9	123.51 (5)
H4—C4—C3	122.4 (6)	O15—C14—O16	120.88 (6)
C10—C9—C8	119.68 (5)	H18A—C18—C6	111.5 (8)
C14—C9—C8	121.91 (5)	H18B—C18—C6	113.2 (8)
C14—C9—C10	118.41 (5)	H18B—C18—H18A	104.1 (13)
H16—O16—C14	110.9 (8)	H18C—C18—C6	114.1 (7)
C12—C11—C10	118.83 (6)	H18C—C18—H18A	106.5 (13)
H11—C11—C10	120.7 (6)	H18C—C18—H18B	106.8 (13)
H11—C11—C12	120.5 (6)		
			
Cl17—C5—C6—C1	179.07 (4)	C6—C1—N7—C8	−142.97 (5)
Cl17—C5—C6—C18	−0.77 (5)	C8—C9—C10—C11	1.22 (7)
Cl17—C5—C4—C3	−179.13 (4)	C8—C9—C14—O16	176.88 (6)
C5—C6—C1—C2	−0.01 (6)	C8—C9—C14—O15	−3.68 (7)
C5—C6—C1—N7	−175.43 (5)	C8—N7—C1—C2	41.71 (8)
C5—C4—C3—C2	0.17 (7)	C8—C13—C12—C11	−0.37 (7)
C6—C1—C2—C3	0.94 (6)	C9—C10—C11—C12	1.17 (8)

### (II) Hydrogen-bond geometry (Å, º)

**Table:**

*D*—H···*A*	*D*—H	H···*A*	*D*···*A*	*D*—H···*A*
N7—H7···O15	1.019 (11)	1.800 (10)	2.6469 (7)	138.0 (8)
O16—H16···O15^i^	0.998 (14)	1.640 (14)	2.6381 (8)	179.0 (12)

Symmetry code: (i) −*x*+2, −*y*+1, −*z*+1.
